# Amazon: between devastation, violence, and threads of hope

**DOI:** 10.1590/0102-311XEN152723

**Published:** 2023-12-22

**Authors:** Luiza Garnelo, Philip Martin Fearnside, Lucas Ferrante

**Affiliations:** 1 Instituto Leônidas & Maria Deane, Fundação Oswaldo Cruz, Manaus, Brasil.; 2 Instituto Nacional de Pesquisas da Amazônia, Manaus, Brasil.; 3 Universidade Federal do Amazonas, Manaus, Brasil.

The global interest in preserving the Amazon, intensified by the need to mitigate global climate change ^1^ ^,^ ^2^, barely affects the daily lives of those who live there. Over the centuries, the predominant economic activities in the Amazon have been based on the cyclical exploitation of natural resources ^3^ ^,^ ^4^. Extractivism, characterized by low technological density and strong anthropization of environments, is carried out with rudimentary tools and intensive allocation of low-skilled, poorly paid labor that is highly exposed to occupational accidents and diseases, and to pathogens present in the environment ^2^ ^,^ ^4^. The extractivist model is not local in scope, but rather efficiently promotes a subordinate integration with the interests and needs of the global economy. This results in an alternation between periods of regional economic recession and growth, depending on the market demand for extracted products and/or depletion of such resources, given the low yield of the activity and the broad impact of extractivist predation.

The military dictatorship encouraged large-scale mineral exploitation, construction of roads, and construction of hydroelectric dams ^2^ that boosted extensive agriculture, cattle ranching and logging, mostly for export ^4^ ^,^ ^5^. The modernization of these production processes was not followed by an equivalent improvement in labor relations, but by increased environmental devastation and violation of the fundamental rights of traditional communities, especially Indigenous peoples ^6^.

During the Jair Bolsonaro’s administration (2019-2022), military forces created smokescreens to cover an exponential increase in the devastation of the Amazon ^7^. The explosion in deforested area from 2019 to 2022 (46,500 km^2^) was 41.4% higher than the average deforestation observed in the previous four years (2015-2018; 27,500 km^2^) ^8^ ^,^ ^9^. This illustrates the pressure of these new cycles on the biome, added to the dismantling of environmental policies. In this context, traditional peoples, such as the Indigenous people, have been strongly affected by the COVID-19 pandemic, which has exacerbated disparities in health indicators due to socioeconomic issues, difficulties in accessing health care, and constant invasions of their land ^9^ ^,^ ^10^.

Environmental devastation also favors the growth of vector-borne diseases such as malaria ^11^ ^,^ ^12^, leishmaniasis, Chagas disease, arboviruses, and other viral pathologies that are still emerging and insufficiently known, but which are of global concern. Infection by pathogens stemming from the devastated forest also provides routes for a zoonotic “leap” capable of altering the endemic-epidemic profile in the region and generating new global epidemics ^13^ ^,^ ^14^.

In the last 10 years, the gross domestic product (GDP) per capita in the Amazon ranged from BRL 25,799.70 in 2010 to BRL 26,054.24 in 2020, values 30.6% lower than GDP per capita of Brazil as a whole. However, for the Amazon, this indicator maintained average real growth of 0.4% per year in the last decade, in contrast to the -0.7% downward trend of the same indicator for the country over the same period ^15^. The employment rate is low in the Amazonian population, as only 34.4% were employed in 2021 ^15^. Although life expectancy has grown, it is still lower than in the other regions (73.2 years in 2021), pari passu with the higher infant mortality rate (14/1,000 live births) compared to the rest of Brazil ^16^ ^,^ ^17^. Official statistics estimate that 45% of the population lives in poverty, an increase of 1.2 percentage points from 2012 to 2021 ^16^. Data from the penultimate census show a dependency ratio of 55.7% for the Northern region, compared to 45.9% for the country as a whole ^17^. These data show that the wealth circulating in the region has not contributed to reducing social inequality, which is even more profound among Indigenous and rural populations in the Amazon.

The Amazonian population is heavily dependent on public health care. However, Scheffer ^18^ showed that the Northern region has a lower ratio of physicians per inhabitant (1.45/1,000) compared to the Federal District, Rio de Janeiro, São Paulo, and Santa Catarina (5.53, 3.77, 3.50, and 3.05/1,000 inhabitants, respectively). Consequently, the proportion of medical consultations in the Northern region is also unfavorable, as in the 12 months prior to the survey there were 2.25 medical consultations per inhabitant/year, with 1.97 consultations per inhabitant without a health insurance plan/year and 3.23 consultations per inhabitant with a health insurance plan/year. On the other hand, the Southeastern region obtained, for the same indicators, 80.8%; 3.43; 3.04; and 4.18, respectively ^18^. These findings show that in the Northern region, inhabitants with sufficient purchasing power to pay for a health insurance plan had almost twice as much access to medical care compared to those who depended exclusively on the Brazilian Unified National Health System (SUS).

There are few social vulnerability indicators available for the region. Usually, the existing data are sparse and come from local studies, hindering a comprehensive assessment of living conditions in the Amazon. One of the sources that analyzes living conditions is the Social Progress Index (IPS), a composite indicator with three evaluation dimensions (basic human needs, foundations for well-being, and opportunities), measured by 45 indicators that scrutinize positive changes in the living conditions of the population of the nine states and 772 municipalities in the Amazon ^19^.

Santos et al. ^19^ found that the IPS 2021 indicated that fulfillment of basic needs of the Amazonian population is well below the results obtained for Brazil as a whole (66.19 against 77.78, respectively). In the same study, opportunities, which for the country as a whole only reach low levels (42.87), are even lower in the Amazon (41.80). More specific topics, such as social inclusion, show mostly low results throughout the region, with several municipalities achieving poor figures for this indicator. The quality of the environment in remote rural municipalities that are not yet significantly affected by environmental devastation is good, but their residents do not enjoy adequate levels of well-being, individual rights, freedom of choice, and social inclusion ^19^.

Violence has been a topic of great concern in discussions about the Amazon. The number of conflicts with fatal consequences has recently escalated and expanded into inland areas, accompanying deforestation and the opening of roads ^20^ ^,^ ^21^. Sources such as the *Atlas of Rural Violence in Brazil* ^22^ point out that the Legal Amazon accounted for 62.4% of agrarian violence in the country in 2020, when 1,576 conflicts were recorded, making that year the one with the highest occurrence of conflicts since 1985. In this context, the growth in the homicide rate stands out, with an increase of 260.3% for the Northern region ^22^.

The major government projects of previous decades generated tax incentives, construction of roads , hydroelectric facilities, and unsustainable colonization, mining, and agricultural initiatives in the Amazon ^23^ ^,^ ^24^. Illicit activities later benefited from these initiatives, taking advantage of the road network, the incipient role of the State, and the limited opportunities for employment and income in the region. As a result, real criminal systems have been structured, bringing together the illegal exploitation of minerals, timber, and fish, associated with drug trafficking, uniting the forest with the cities to guarantee a monopoly on the wholesale trade of drugs and other illegal products ^25^. In other words, crime also perceives the global strategic relevance of the Amazon.

This broad set of vulnerabilities coexists with initiatives that seek to produce sustainable income-generating alternatives, empower local communities and groups to exercise political power, and reduce the current inequities. Among these, it is important to highlight the work of indigenous organizations that have been leading struggles for territorial management and the expansion of income-generating alternatives, and confronting the State in defense of their lives and access to health care, as was the case during the COVID-19 pandemic ^26^. These initiatives demonstrate that solutions to the Amazon’s problems require the empowerment and qualification of political agents from civil society in the region, instead of solutions imposed from outside, which have been repeated for decades without positive results.

The scale of the problems afflicting the Amazon demands an intersectoral policy, supported by a firm alliance of people and institutions from inside and outside the region, seeking to combine social justice with environmental preservation, combating corruption and crime, as well as promoting improvement in health and education indicators. These efforts alone will be insufficient to face the challenge posed by the current situation, unless they are combined with ongoing initiatives to reverse the economic model operating in the region and establish a sustainable economy that is capable of extending job and income opportunities to inland populations and creating alternatives for existence that are not dependent on the predatory extractivism currently in force.
